# Network–Polymer–Modified Superparamagnetic Magnetic Silica Nanoparticles for the Adsorption and Regeneration of Heavy Metal Ions

**DOI:** 10.3390/molecules28217385

**Published:** 2023-11-01

**Authors:** Yaohui Xu, Yuting Li, Zhao Ding

**Affiliations:** 1 Laboratory for Functional Materials, School of New Energy Materials and Chemistry, Leshan Normal University, Leshan 614000, China; xuyaohui1986@163.com; 2 Leshan West Silicon Materials Photovoltaic New Energy Industry Technology Research Institute, Leshan 614000, China; 3 The State Key Laboratory of Refractories and Metallurgy, Institute of Advanced Materials and Nanotechnology, Wuhan University of Science and Technology, Wuhan 430081, China; lyt@wust.edu.cn; 4 College of Materials Science and Engineering, National Engineering Research Center for Magnesium Alloys, Chongqing University, Chongqing 400044, China

**Keywords:** nanoparticles, magnetic, surface modification, adsorption, regeneration, heavy metal ion

## Abstract

Superparamagnetic magnetic nanoparticles (MNPs, Fe_3_O_4_) were first synthesized based on a chemical co–precipitation method, and the core–shell magnetic silica nanoparticles (MSNPs, Fe_3_O_4_@SiO_2_) were obtained via hydrolysis and the condensation of tetraethyl orthosilicate onto Fe_3_O_4_ seed using a sol–gel process. Following that, MSNPs were immobilized using a three–step grafting strategy, where 8-hloroacetyl–aminoquinoline (CAAQ) was employed as a metal ion affinity ligand for trapping specific heavy metal ions, and a macromolecular polymer (polyethylenimine (PEI)) was selected as a bridge between the surface hydroxyl group and CAAQ to fabricate a network of organic networks onto the MSNPs’ surface. The as–synthesized MSNPs–CAAQ nanocomposites possessed abundant active functional groups and thus contained excellent removal features for heavy metal ions. Specifically, the maximum adsorption capacities at room temperature and without adjusting pH were 324.7, 306.8, and 293.3 mg/g for Fe^3+^, Cu^2+^, and Cr^3+^ ions, respectively, according to Langmuir linear fitting. The adsorption–desorption experiment results indicated that Na_2_EDTA proved to be more suitable as a desorbing agent for Cr^3+^ desorption on the MSNPs–CAAQ surface than HCl and HNO_3_. MSNPs–CAAQ exhibited a satisfactory adsorption capacity toward Cr^3+^ ions even after six consecutive adsorption–desorption cycles; the adsorption efficiency for Cr^3+^ ions was still 88.8% with 0.1 mol/L Na_2_EDTA as the desorbing agent. Furthermore, the MSNPs–CAAQ nanosorbent displayed a strong magnetic response with a saturated magnetization of 24.0 emu/g, and they could be easily separated from the aqueous medium under the attraction of a magnet, which could facilitate the sustainable removal of Cr^3+^ ions in practical applications.

## 1. Introduction

Heavy metal ions (HMIs) can easily spread through the food chain and seriously threaten human health [1,2,3], as well as negatively influencing the ecological environment of sustainable development [4,5,6]. Chromium (Cr^3+^), arsenic (As^3+^), cadmium (Cd^2+^), mercury (Hg^2+^), and plumbum (Pd^2+^) ions were considered to be the five most toxic HMIs, with intense toxicity and the tendency to cause serious diseases even at very low concentrations [7]. Therefore, HMI pollution in water caused by modern industry is an important issue due to its toxicity, complex components, and non–biodegradability. Usually, HMI pollution in water has the characteristics of a large pollution area, so it is difficult to enrich HMIs in water using extraction methods. This has been presented as a simple and effective method to fix HMIs in situ to reduce their toxicity in water due to the obvious repair effect, simple construction technology, short construction period, reasonable repair costs, and other characteristics. Among the technologies used to cure or stabilize HMIs in water, the adsorption method is the most economical and effective method [8,9,10]. Reasonable and efficient adsorbents are the key to determining the feasibility of the purification and recovery of HMIs in water by adsorption. The primary adsorbents used in wastewater treatment are categorized as carbon [11], silica [12], zeolite [13], metal–based [14] nano–adsorbents, and magnetic nanoparticles [15]. In addition to the above main classes of adsorbents, several economical bio–adsorbents have been produced from agricultural waste, food waste, cellulose waste, and industrial by–products [16,17].

The non–magnetic nanosorbents are easy to lose in the process of use, which is not conducive to recovery. In any case, exhausted non–magnetic nano–adsorbents have to be removed from the liquid, and magnetic extraction appears to be the most frequently adopted solution [10]. Therefore, magnetic nanomaterials, especially superparamagnetic nanomaterials, have received extensive attention from researchers. Magnetic nanoparticles (MNPs, Fe_3_O_4_) are an excellent candidate nanosorbent because of their small size effect and apparent superparamagnetism [18]. The greatest strength of MNPs is that they are easily attracted to the target zone under the guidance of an external magnetic field [19,20,21,22,23]. An inconvenient disadvantage of MNPs is that they are prone to oxidative degradation when directly exposed to the environment. In order to reduce the instability of MNPs, a core–shell structure of magnetic silica nanoparticles (MSNPs) is proposed, in which the MNPs, as the core, can facilitate targeted control and recyclable separation under an applied magnetic field, while the silica (SiO_2_), as the shell, can prevent the magnetic core from oxidation and corrosion [24].

Surface modification proved to be an effective way to improve the adsorption performance of nanosorbents. At present, many studies are focused on the surface modification of MSNPs to improve their adsorptive capacity by grafting the end–reactive functional groups. Generally, the grafted organic molecules, such as iminodiacetic acid, (3–aminopropyl)triethoxysilane, N–(trimethoxysilylpropyl)ethylenediamine triacetic acid, and 3–mercaptopropyltrimethoxysilane, had a distinct affinity for combining with Zn^2+^ [25], Cu^2+^ [26], Pb^2+^ [27], Cd^2+^ [28], and Hg^2+^ [29] ions. However, the single–stranded grafting of small organic molecules still cannot meet the actual application demand. On this basis, polymer grafting has been proposed to increase the amount of end–reactive functional groups, which yields MSNPs/polymers with excellent adsorptive features [30,31,32]. Despite this progress in the synthesis of MSNPs–based absorbents, it is still challenging to further improve their adsorptive capacity since further increases in the amount of ligands or the introduction of large macromolecules are restrained by the steric hindrance, limiting their actual applications.

**Figure 1:**
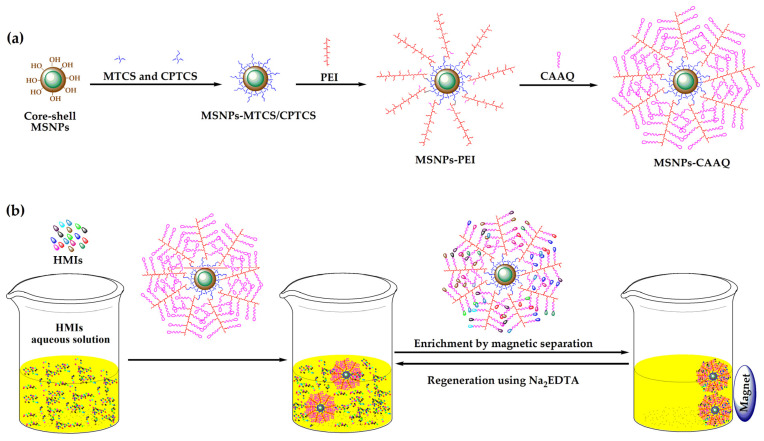
(**a**) Synthesis scheme of MSNPs–CAAQ nanocomposites; (**b**) schematic diagram of HMIs adsorption and magnetic separation.

In this regard, we proposed network polymer (8–chloroacetyl–aminoquinoline, CAAQ)–modified MSNPs to remove HMIs using a three–step grafting strategy, as shown in Figure 1a. In the design of this network structure on MSNPs’ surfaces, the selected macromolecular polymer was key. Polyethylenimine (PEI) possessed numerous branched chains of –NH_2_ groups, which formed the framework of a network structure. MSNPs were like a tree, and CAAQ was the final fruit (end–active group), while PEI was like a branch with rich twigs (–NH_2_ group), providing numerous sites for the growth of the final fruit. The network design could effectively increase the quantity of active functional groups in the limited space and thus improve their adsorption capacity. The key process of this synthesis was to achieve a controllable bridge of MTCS/CPTCS and PEI, allowing for more CAAQ to become immobilized onto MSNPs. Moreover, the removal and regeneration of HMIs by MSNPs–CAAQ from aqueous solutions was investigated, as shown in Figure 1b.

## 2. Results and Discussion

### 2.1. Characterizations of MNPs and MSNPs

**Figure 2:**
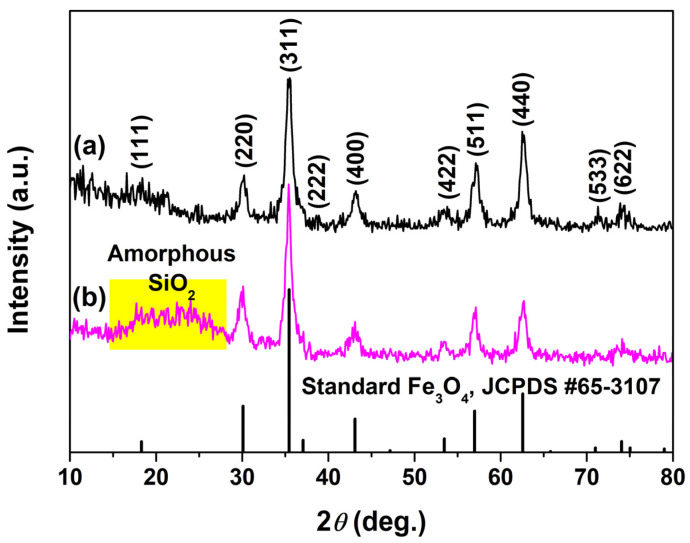
XRD spectra of (**a**) MNPs and (**b**) MSNPs contrasted with the standard Fe_3_O_4_ (JCPDS No. 65–3107) pattern.

Figure 2a,b show the XRD patterns of the as–synthesized MNPs and MSNPs, respectively. The XRD pattern of MNPs in Figure 2a displays several well–resolved diffraction peaks that could match well with the (111), (220), (311), (222), (400), (422), (511), (440), (533) and (622) planes of cubic spinel Fe_3_O_4_ (JCPDS No. 65–3107). All crystal planes could be identified, indicating that the obtained Fe_3_O_4_ crystal was complete, and the average grain size of Fe_3_O_4_ was calculated with a value of about 9.0 nm according to Scherrer’s formula. In addition to the diffraction peaks of the cubic spinel Fe_3_O_4_ phase, there was a weak and broad peak at 2*θ* = 15~28° (yellow area in Figure 2b), which was consistent with the amorphous SiO_2_ phase. Compared to the XRD diffraction intensity of MNPs in Figure 2a, that of MSNPs in Figure 2b was slightly reduced, further implying that the amorphous SiO_2_ was coated onto the surface of Fe_3_O_4_ seeds. Further analysis of the core–shell structure of MSNPs was conducted by TEM analysis, as discussed later.

**Figure 3:**
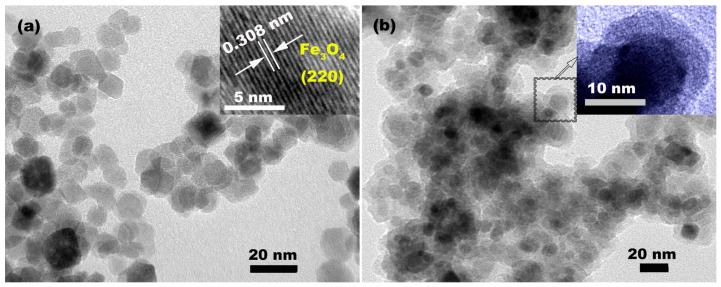
(**a**) TEM image of MNPs (inset is an HRTEM image of MNPs); (**b**) TEM image of MSNPs (inset is an amplified TEM image of MSNPs).

TEMs were employed to characterize the morphology, size, and microstructure of MNPs and MSNPs. The TEM image in Figure 3a demonstrates that the as–synthesized MNPs exhibited a uniform size of ~10 nm. Moreover, we could observe that these particles had the same direction of lattice fringes with an interplanar spacing of 0.308 nm from the high–resolution TEM (HRTEM) image in Figure 3a’s inset, fitted with (220) in cubic spinel Fe_3_O_4_, which proves that the as–synthesized Fe_3_O_4_ was a single–crystalline structure. After coating with SiO_2_, the particle size increased to ~21 nm from ~10 nm, according to the TEM image in Figure 3b. Furthermore, the obvious core–shell structure with a core size of ~10 nm and a shell thickness of ~5.5 nm could be clearly observed from the amplified TEM image in Figure 3b’s inset. The XRD data in Figure 2 and TEM data in Figure 3 could prove that MNPs with a single–crystal structure can be synthesized using the chemical co–precipitation method, while MSNPs with a core–shell structure can be obtained through the hydrolysis and condensation of TEOS on a single–crystal Fe_3_O_4_ seed.

### 2.2. Characterizations of MSNPs–CAAQ

**Figure 4:**
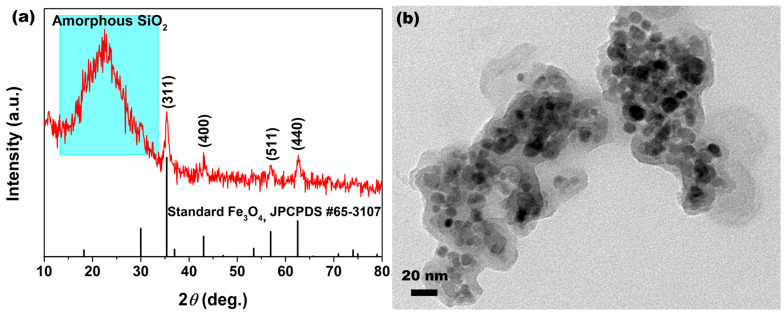
(**a**) XRD spectrum contrasted with standard Fe_3_O_4_ (JCPDS NO. 65–3107) pattern and (**b**) TEM image of MSNPs–CAAQ.

Figure 4a shows the XRD spectrum of as–synthesized MSNPs–CAAQ. In Figure 4a, the broad peak at 2*θ* = ~22.5° or other sharp little peaks at 2*θ* > ~33.0° imply the existence of at least two kinds of phases in the MSNPs–CAAQ samples. The broad peak at 2*θ* = ~22.5° could be attributed to some phases with an amorphous state, while the several sharp little peaks at 2*θ* > ~33.0° were indexed to the (311), (400), (511), and (440) planes of the standard Fe_3_O_4_ (JCPDS No. 65–3107) pattern, indicating the presence of the cubic spinel Fe_3_O_4_ phase. Compared with the XRD patterns of MNPs and MSNPs in Figure 2, the diffraction peak intensity of the Fe_3_O_4_ phase was reduced dramatically, while the broad featureless peak of SiO_2_ was clearly increased, becoming even stronger than that of the Fe_3_O_4_ phase, indicating that organic molecules were coated on the MSNPs’ surface after modification. Figure 4b shows the TEM image of MSNPs–CAAQ. In Figure 4b, an extra amorphous coating can be observed around the particles, further implying successful CAAQ modification.

**Figure 5:**
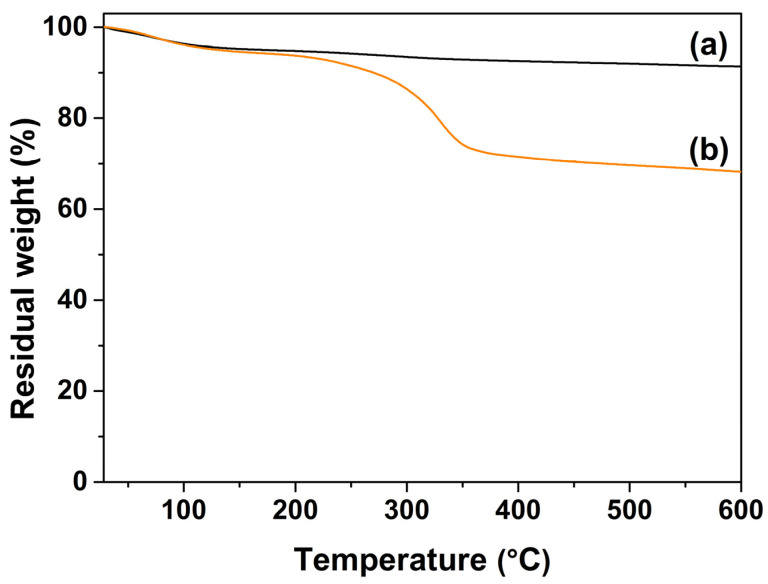
TG curves of (a) MSNPs and (b) MSNPs–CAAQ.

The thermal decomposition behaviors of MSNPs and MSNPs–CAAQ were determined using a TG instrument at a heating rate of 10 °C/min under a flow of air within the temperature range of 25 to 600 °C. For the TG curve of MSNPs in Figure 5a, a relative weight loss of 8.6% was detected over the entire temperature range, which was probably caused by the evaporation of adsorbed water and the decomposition of hydroxyl groups (–OH) on the MSNPs’ surface, while a far higher weight loss of 31.7% over the entire temperature range was observed for MSNPs–CAAQ in Figure 5b, which could mainly be attributed to the evaporation of adsorbed water and the decomposition of the grafted organic molecules layer, including MTCS, CPTCS, PEI, and CAAQ. Below ~150 °C, the TG curves of MSNPs and MSNPs–CAAQ almost coincided, indicating that the water content on the surface of MSNPs and MSNPs–CAAQ was basically the same. Therefore, the weight percentage of grafted organic molecules on MSNPs’ surface could be calculated by subtracting the weight loss of MSNPs from that of MSNPs–CAAQ, and the value was ~23.1%.

**Figure 6:**
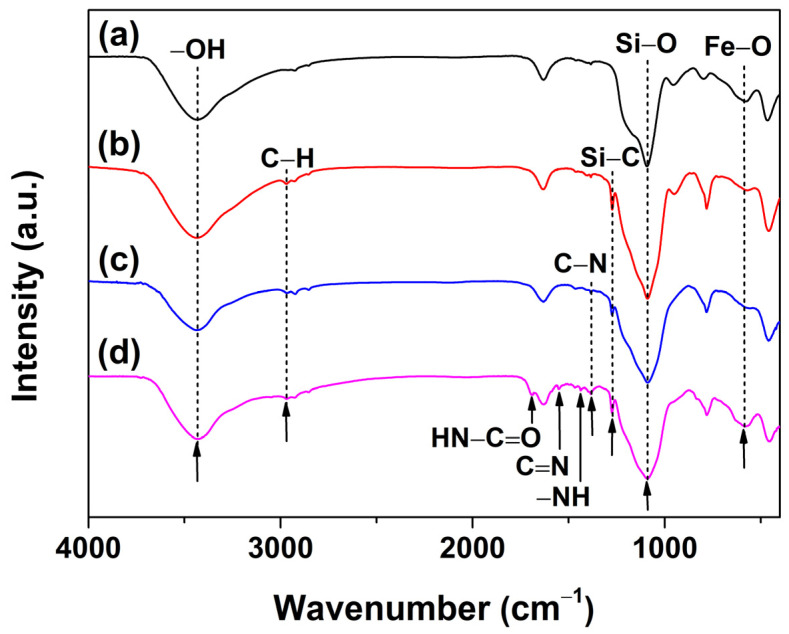
FTIR spectra of (a) MSNPs, (b) MSNPs–MTCS/CPTCS, (c) MSNPs–PEI, and (d) MSNPs–CAAQ.

To determine the organic composition of MSNPs’ surfaces, FTIR and XPS analyses were employed. Figure 6a–d shows the FTIR spectra of MSNPs, MSNPs–MTCS/CPTCS, MSNPs–PEI, and MSNPs–CAAQ, respectively. For all FTIR spectra in Figure 6, there were several apparent absorption peaks at 3433, 1088, and 584 cm^−1^, corresponding to the stretching of –OH [33], Si–O [34], and Fe–O [35] bonds, respectively. This further demonstrated the successful synthesis of Fe_3_O_4_/SiO_2_ composites with numerous –OH groups, which was consistent with the XRD analysis results in Figure 2. Compared with the FTIR spectrum of MSNPs in Figure 6a, two new absorption peaks at 2970 cm^−1^ (C–H bond) and 1274 cm^−1^ (Si–C bond) [36] were observed. For the FTIR spectrum of MSNPs–MTCS/CPTCS in Figure 6b. In addition to the absorption peaks of C–H and Si–C bonds, the FTIR spectrum of MSNPs–PEI presented in Figure 6c showed a new peak at 1392 cm^−1^ [37], ascribed to the stretching of the C–N bond. In addition to the above–mentioned absorption peaks in Figure 6a–c, there were also three new absorption peaks at 1695, 1551, and 1437 cm^−1^ for the FTIR spectrum of MSNPs–CAAQ in Figure 6b, ascribed to the stretching of HN–C=O, C=N, and –NH bonds [38], respectively. This indicates that MTCS/CPTCS, PEI, and CAAQ were successfully grafted onto the MSNPs’ surface.

**Figure 7:**
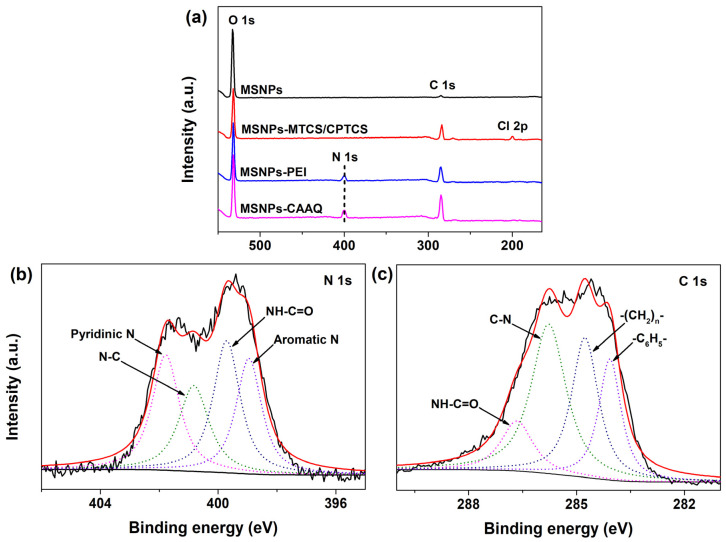
XPS wide scan of (**a**) MSNPs, MSNPs–MTCS/CPTCS, MSNPs–PEI, and MSNPs–CAAQ; (**b**) C 1s and (**c**) N 1s core levels of MSNPs–CAAQ.

Figure 7a shows the XPS spectra of MSNPs, MSNPs–MTCS/CPTCS, MSNPs–PEI, and MSNPs–CAAQ. By comparing the XPS spectrum of MSNPs, that of MSNPs–MTCS/CPTCS showed a clear new peak in the Cl signal at ~200.0 eV, which probably derived from the MTCS and CPTCS grafted onto the MSNPs’ surface. The XPS spectra of MSNPs–PEI and MSNPs–CAAQ both showed a clear new peak in N element at ~400.0 eV, which probably derived from the nitrogenous organic matter grafted onto MSNPs’ surfaces, including PEI and CAAQ. In addition, the absence of a Cl signal for the XPS spectrum of MSNPs–PEI suggested a successful dechlorination coupling reaction between MSNPs–MTCS/CPTCS and the PEI molecule. Moreover, the detailed N 1s core–level spectrum of MSNPs–CAAQ could be curve–fitted into four peak components, as shown in Figure 7b. The binding energies at 401.8, 400.8, 399.7, and 398.9 eV could be attributed to pyridinic N species, N–C species, HN–C=O species, and aromatic N species, respectively [39,40,41]. Figure 7c also shows the XPS spectrum of C 1s core levels for MSNPs–CAAQ. From Figure 7c, the C 1s core–level spectrum is shown to present four peaks with binding energies of 286.7, 285.8, 284.8, and 284.1 eV, attributable to the NH–C=O, C–N, –(CH_2_)_n_–, and –C_6_H_5_– species, respectively [42,43,44,45]. This further confirms that the CAAQ–modified MSNPs were successfully yielded using the three–step grafting method (see Figure 1a), involving MTCS/CPTCS co–modification, PEI grafting, and CAAQ grafting.

### 2.3. Magnetic Analysis of MNPs, MSNPs and MSNPs–CAAQ

**Figure 8:**
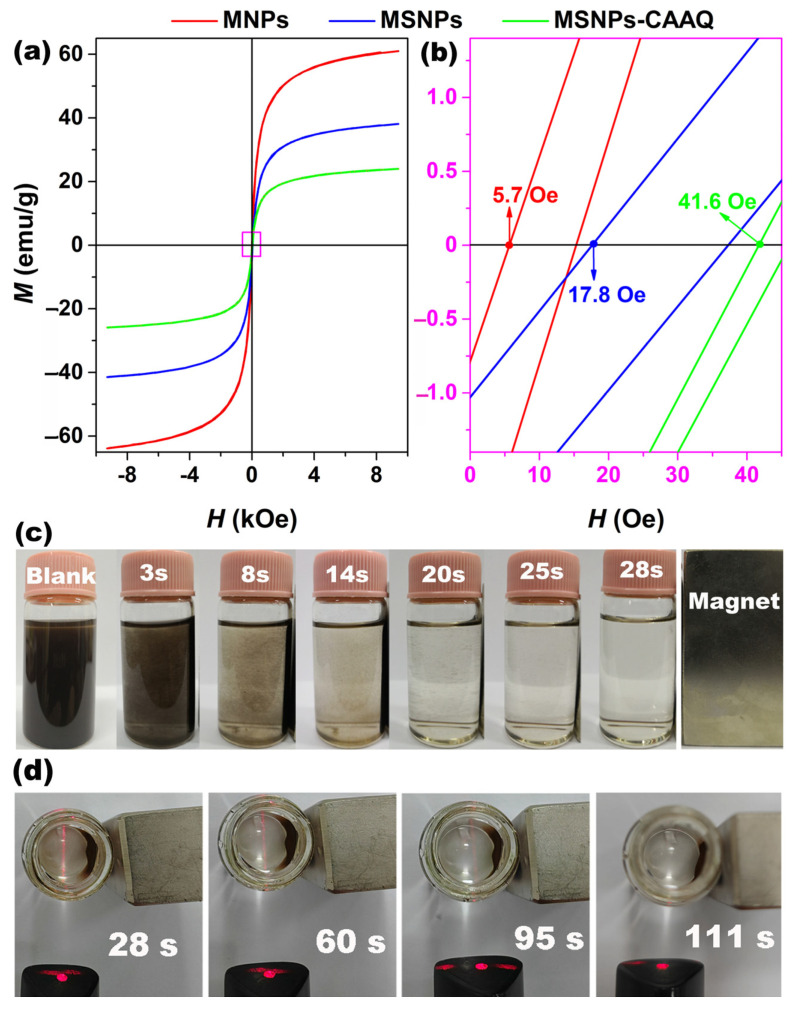
(**a**) Room–temperature magnetic hysteresis loops of MNPs, MSNPs, and MSNPs–CAAQ; (**b**) the amplified curves around the coercive value (the area inside the pink box in Figure 8a); (**c**) time–dependence of the magnetic response behavior of MSNPs–CAAQ suspension after adding an external magnet to the right side of the bottle; (**d**) the clear liquid of the Tyndall effect over time.

The magnetic properties were measured using a vibrating sample magnetometer. As displayed in Figure 8a, the saturated magnetizations (*M*) of MNPs, MSNPs, and MSNPs–CAAQ were 61.0, 38.1, and 24.0 emu/g, respectively. Compared with MNPs, the reduced *M* values for MSNPs and MSNPs–CAAQ were mainly attributed to the increased weight of non–magnetic layers, including amorphous SiO_2_ and grafted organic molecules [46]. Figure 8b shows the magnified curve of the surrounding origin in Figure 8a (the area inside the pink box of Figure 8a). All three samples of MNPs, MSNPs, and MSNPs–CAAQ could be regarded as superparamagnetic with negligible hysteresis. This suggests that MNPs encapsulated in the SiO_2_ shell could preserve their superparamagnetism. In practical applications, it is extremely important that the magnetic carriers or supports exhibit rapid responsiveness under an applied magnetic field without retaining any magnetism after withdrawing the applied magnetic field. Moreover, Figure 8c shows the time–dependence of the magnetic response behavior of MSNPs–CAAQ suspension by adding an external magnet to the right side of the bottle. In Figure 8c, these particles are shown to be well dispersed in an aqueous medium without a magnet (Blank in Figure 8c), and they were easily separated from the solution under the attraction of the magnet. The solution appeared clear and transparent within 28 s. However, the Tyndall effect can be observed by the naked eye in Figure 8d, indicating that some MSNPs–CAAQ nanoparticles were still floating in the aqueous solution. As the magnetic separation continued, the Tyndall effect disappeared at 111 s, indicating that the solid–liquid separation was complete. This is a potential drawback of the MSNPs–CAAQ nano–adsorbent due to its low saturation magnetization, which risks leaving behind some nanoparticles that were uncaught by the applied magnetic field. In order to minimize this shortcoming, it was necessary to extend the operation time of the applied magnetic field.

### 2.4. Adsorption Characteristics

**Figure 9:**
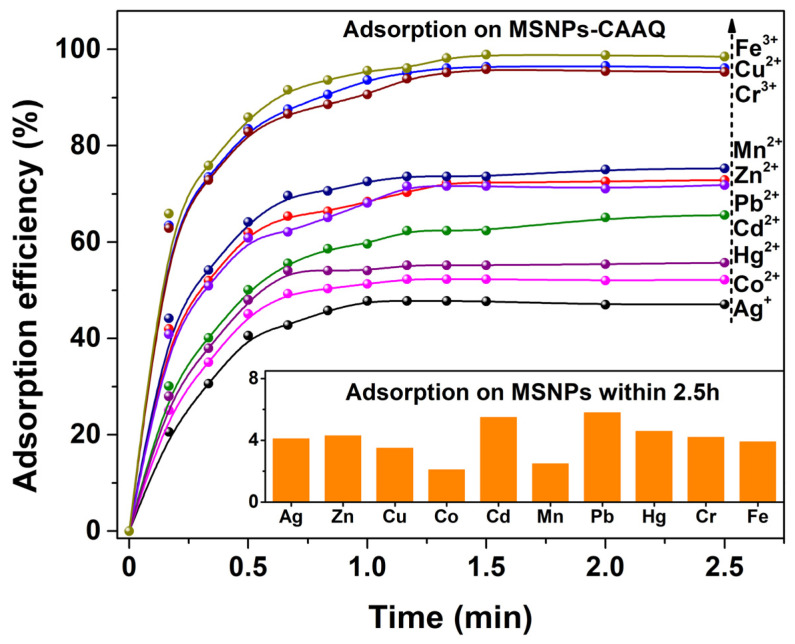
Adsorption profiles onto MSNPs–CAAQ for HMIs (inset shows the adsorption efficiencies within 2.5 h of MSNPs without CAAQ modifications for HMIs). (Adsorbent dose was 1.0 g/L; adsorbate dose was 200 mg/L; *V* = 50 mL; reaction temperature was 298.15 K; no pH pre–adjustments).

The adsorption capacity of MSNPs–CAAQ for the selected HMIs, including Ag^+^, Zn^2+^, Cu^2+^, Co^2+^, Cd^2+^, Mn^2+^, Pb^2+^, Hg^2+^, Cr^3+^ and Fe^3+^ ions, was evaluated at room temperature without pH pre–adjustments. Figure 9 shows the time–dependence of the adsorption profiles of the selected HMIs onto MSNPs–CAAQ composites. Figure 9 shows that the as–synthesized MSNPs–CAAQ exhibited strong and rapid adsorption features for HMIs within the first 1.0 h, and the adsorption process was mostly completed within 1.5 h of the reaction. For Fe^3+^, Cu^2+^ and Cr^3+^ ions, the adsorption efficiencies within 1.5 h could reach 98.9%, 96.4%, and 95.9%, respectively. Moreover, MSNPs–CAAQ exhibited universal adsorption capacities for Zn^2+^, Mn^2+^ and Pb^2+^ ions, with adsorption efficiencies of over 70% within 1.5 h. The sequence and values of the adsorption efficiency within 2.5 h were as follows: Fe^3+^ (98.5%) > Cu^2+^ (96.1%) > Cr^3+^ (95.3%) > Mn^2+^ (75.3%) > Zn^2+^ (72.9%) > Pb^2+^ (71.8%) > Cd^2+^ (65.6%) > Hg^2+^ (55.7%) > Co^2+^ (52.2%) > Ag^+^ (47.1%). As a comparison, the adsorption efficiency of MSNPs without CAAQ modifications was also tested and is shown in the inset of Figure 9. As observed, the adsorption efficiencies of MSNPs within 2.5 h were below 6.0% for all HMIs under the same conditions, indicating that the absorption of HMIs was mainly attributed to the grafted CAAQ rather than MSNPs. The high adsorption efficiencies of MSNPs–CAAQ could be due to the abundant adsorption sites from the CAAQ network that were transferred onto the MSNPs’ surface, which also demonstrates the superiority of our design.

**Figure 10:**
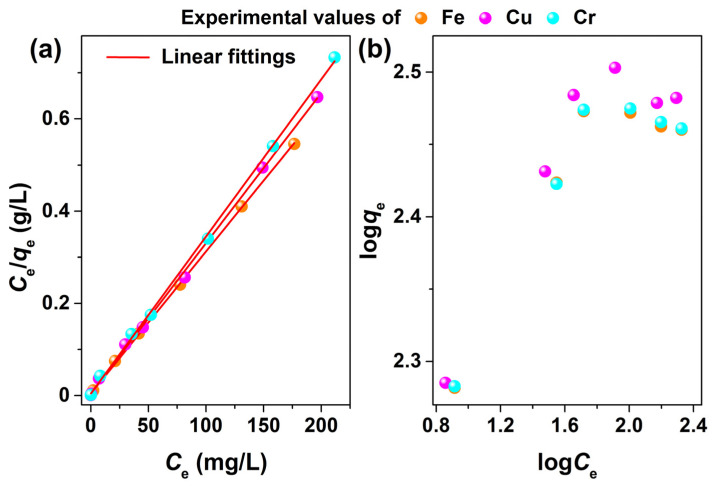
(**a**) Langmuir linear fittings and (**b**) Freundlich plots of Fe^3+^, Cu^2+^, and Cr^3+^ ions onto MSNPs–CAAQ.

The experimental data of the adsorption of Fe^3+^, Cu^2+^, and Cr^3+^ ions onto MSNPs–CAAQ were analyzed by the Langmuir isotherm model and Freundlich isotherm model; their Langmuir linear fittings and Freundlich plots are shown in Figure 10a,b, and the corresponding Langmuir and Freundlich parameters that were calculated are listed in Table 1. From Figure 10a, it is shown that the Langmuir isotherm model was a good fit for modeling the adsorption of Fe^3+^, Cu^2+^, and Cr^3+^ ions onto the MSNPs–CAAQ surface. However, the experimental data considerably deviated from the Freundlich model in Figure 10b, suggesting that it was not appropriate to use the Freundlich model to predict the adsorption isotherms of Fe^3+^, Cu^2+^, and Cr^3+^ ions onto MSNPs–CAAQ. As observed in Table 1, the Langmuir isotherm model had high correlation coefficients (*R*^2^ > 0.9980), implying that the adsorption of Fe^3+^, Cu^2+^, and Cr^3+^ ions could be better described by the Langmuir model. The saturated adsorption amounts (*q*_m_) of Fe^3+^, Cu^2+^, and Cr^3+^ ions were 324.7, 306.8, and 293.3 mg/g, respectively, according to the Langmuir linear fitting. Moreover, a comparison of the maximum adsorption capacities of Fe^3+^, Cu^2+^, and Cr^3+^ ions onto other adsorbents is shown in Table 2 [47,48,49,50,51]. Except for the adsorption of Cu^2+^ and Cr^3+^ ions onto EDTA–inspired polydentate hydrogels, the adsorption capacities were significantly higher for the MSNPs–CAAQ sorbents used in this study.

**Figure 11:**
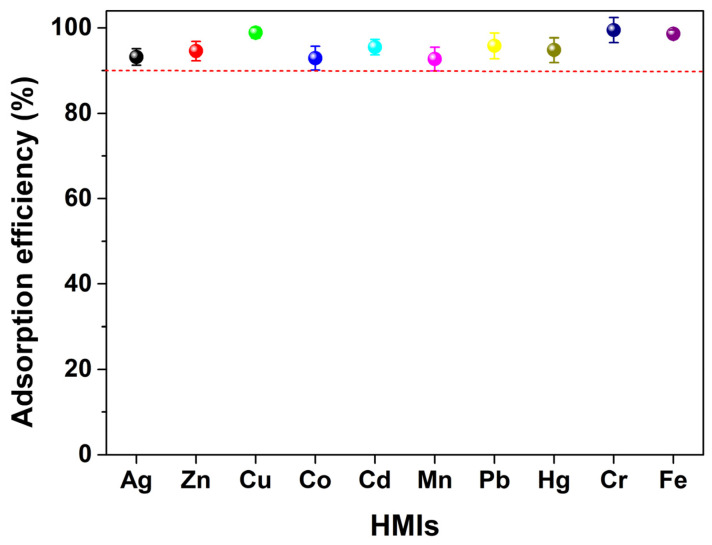
Adsorption efficiency of MSNPs–CAAQ for mixed–HMIs solution. (Mixed HMIs contained Ag^+^, Zn^2+^, Cu^2+^, Co^2+^, Cd^2+^, Mn^2+^, Pb^2+^, Hg^2+^, Cr^3+^, and Fe^3+^ ions; the mass of each HMI was 20 mg; MSNPs–CAAQ was 0.5 g; *V* = 500 mL; *t* = 1.5 h; reaction temperature was 298.15 K; no pH pre–adjustments). (Note: the different color ball represent was the adsorption efficiency of MSNPs–CAAQ for different HMIs in the mixed solution).

In actual industrial wastewater, the coexistence of multiple HMIs is common, so it was necessary to investigate the influence of competing ions on adsorption. An adsorption experiment in a mixed–HMIs solution was also carried out, in which the mass of each HMI was 20 mg, the mass of the MSNPs–CAAQ adsorbent was 0.5 g, and the total volume of solution was 500 mL. The adsorption efficiency of the matrixed HMIs is shown in Figure 11. As observed, MSNPs–CAAQ exhibited universal and excellent adsorption capacities for all HMIs; the adsorption efficiencies could reach over 92.0% within 1.5 h, mainly due to the large adsorption capacity of the MSNPs–CAAQ adsorbent and the low initial concentration of each HMI in the mixed solution. It could be concluded from the above adsorption experiments that MSNPs–CAAQ is suitable as an adsorbent for the removal of HMIs, especially when the concentration of HMIs is low. In view of the strong toxicity of the Cr^3+^ ion, the adsorption characteristics of Cr^3+^ ions on MSNPs–CAAQ will be explored in the following research.

### 2.5. Effect of pH on Adsorption

**Figure 12:**
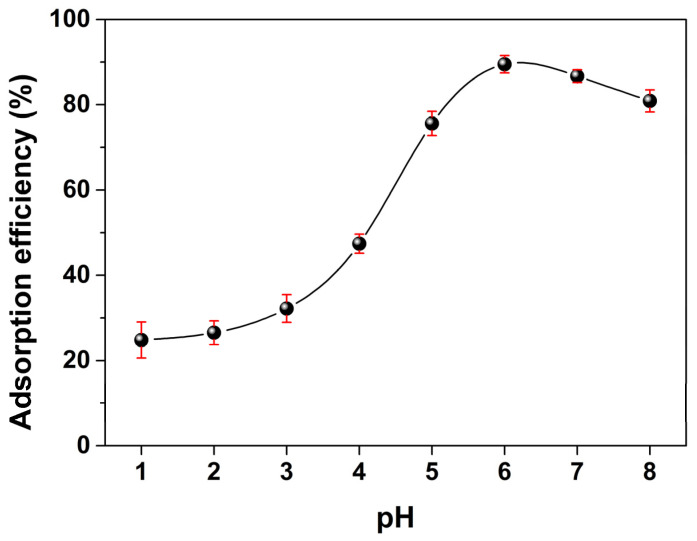
Effect of pH on the adsorption of Cr^3+^ ions by MSNPs–CAAQ. (The adsorbent dose was 0.05 g; the initial concentration and volume of Cr^3+^ ions were 250 mg/L and 50 mL; *t* = 1.5 h; the reaction temperature was 298.15 K).

Figure 12 shows the adsorption efficiencies of MSNPs–CAAQ at various pH values by plotting the normalized adsorption efficiency at different pH values. The initial concentration and volume of Cr^3+^ ions were 250 mg/L and 50 mL; the mass of MSNPs–CAAQ was 1.0 g/L; and the pH value (1~8) of the aqueous solution was adjusted using HCl and NaOH aqueous solutions. Figure 12 shows that the pH value of the aqueous solution has a great influence on the adsorption efficiency of MSNPs–CAAQ. Below pH = 2.0, the adsorption efficiency of Cr^3+^ ions was very low, which could be attributed to the electrostatic repulsion between the positively charged Cr^3+^ ions and the protonated surface of MSNPs–CAAQ under strong acidic conditions. However, the adsorption efficiency increased steeply starting from pH = 3.0 and reached its maximum at around pH = 6.0, which could be attributed to the formation of a metal complex on the MSNPs–CAAQ surface. The decrease at around pH = 7.0 and 8.0 could be attributed to the dissociation of the loaded Cr^3+^ ions on the MSNPs–CAAQ surface under strong basic conditions.

### 2.6. Effect of Temperature on Adsorption

**Figure 13:**
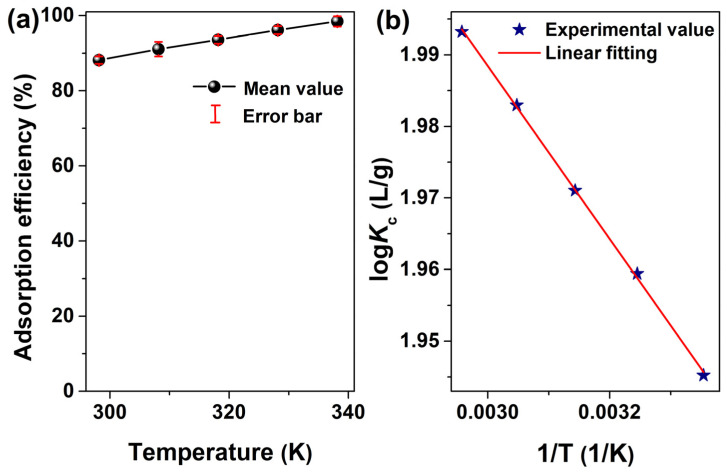
(**a**) Effects of temperature on the adsorption efficiency of the Cr^3+^ ion on MSNPs–CAAQ; (**b**) experimental data of adsorption of the Cr^3+^ ion at a set temperature of 298.15, 308.15, 318.15, 328.15, and 338.15 K on MSNPs–CAAQ fitted using the Van’t Hoff equation. (Adsorbent dose was 1.0 g/L; adsorbate dose was 300 mg/L; *V* = 50 mL; *t* = 1.5 h; no pH pre–adjustments).

For chemical adsorption, the degree of adsorption for the adsorbent would increase with the increase in adsorption reaction temperature, and the opposite rule would hold for physical absorption. Therefore, the effects of temperature on the adsorption of Cr^3+^ ions were investigated in the range of 298.15~338.15 K in this work. In Figure 13a, the adsorption efficiency of the Cr^3+^ ion on MSNPs–CAAQ is shown to slowly increase with an increase in temperature, which implies the possibility of a chemical–reaction–led adsorption process. Moreover, these experimental data on adsorption were fitted using the Van’t Hoff equation, as shown in Figure 13b. Moreover, thermodynamic parameters such as ∆*H*^0^ and ∆*S*^0^ were calculated and are summarized in Table 3. In Table 3, a highly associated correlation coefficient (*R*^2^) of 0.9993 was obtained, suggesting the reliability of the thermodynamic fitting result in this work. The positive value of ∆*H*^0^ (2.31 KJ/mol) indicates that the adsorption reaction was endothermic, while the positive value of ∆*S*^0^ (45.00 J/mol·K) implies that the disorder and randomness at the solid solution interface of the Cr^3+^ ion with MSNPs–CAAQ increase during the adsorption process.

### 2.7. Adsorption Kinetics

**Figure 14:**
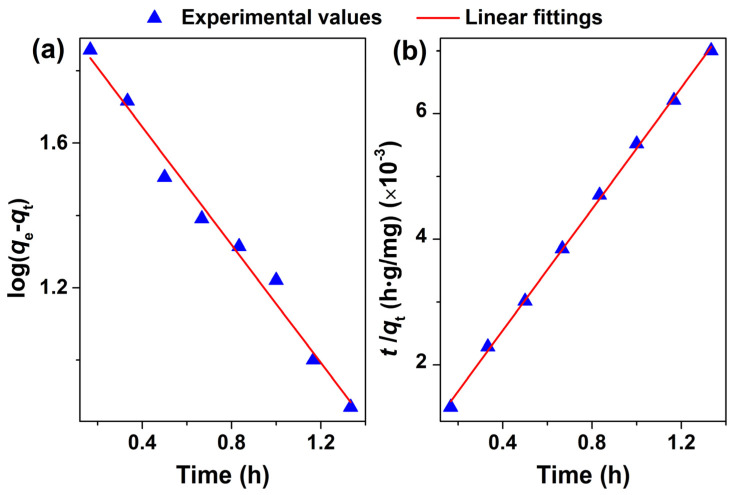
Linear fittings by (**a**) pseudo–first–order and (**b**) pseudo–second–order kinetic models for the adsorption of Cr^3+^ ions onto MSNPs–CAAQ.

**Figure 15:**
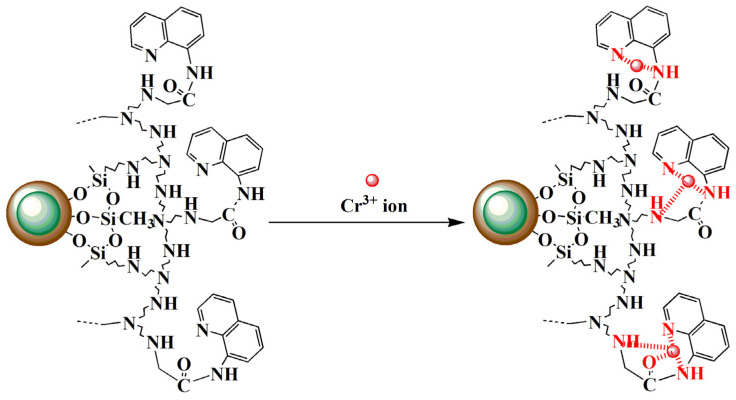
The proposed coordination modes between MSNPs–CAAQ and Cr^3+^ ions.

The sorption kinetics of the Cr^3+^ ion onto MSNPs–CAAQ were tested with the pseudo–first–order and pseudo–second–order kinetic models by plotting log(*q*_e_–*q*_t_) versus *t* (Figure 14a) and plotting t/*q*_t_ versus *t* (Figure 14b), and the kinetic parameters calculated by fitting with these two models are listed in Table 4. In Table 4, the pseudo–second–order equation showed a higher correlation coefficient (*R*^2^ = 0.9989) than that of the pseudo–second–order equation (*R*^2^ = 0.9841). Moreover, the adsorption values calculated at equilibrium (*q*_e,cal_, 206.61 mg/g) from the pseudo–second–order kinetic model were much closer to those of the experimental model (*q*_e,exp_, 191.8 mg/g). Therefore, the pseudo–second–order model was more suitable for describing the adsorption kinetics of the Cr^3+^ ion onto MSNPs–CAAQ, indicating that the chemisorption was the rate–controlling step during the attachment process. Furthermore, the three plausible interaction modes of MSNPs–CAAQ with the Cr^3+^ ion that were proposed are shown in Figure 15, in which the Cr^3+^ ions were selectively coordinated with carbonyl “O” and three “N” atoms. Similar coordination modes have been reported previously [55,56,57].

### 2.8. Desorption and Reusability

**Figure 16:**
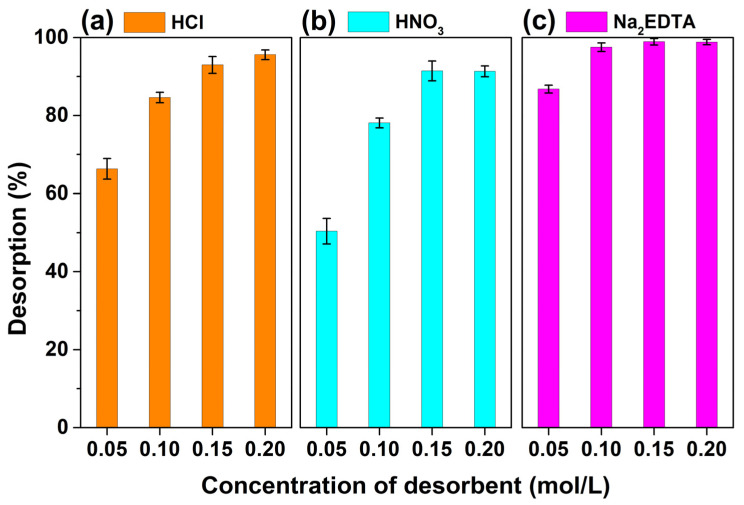
Effect of some desorbing afents (**a**) HCl, (**b**) HNO_3_, and (**c**) Na_2_EDTA with different concentrations (0.05~0.20 mol/L) on the desorption of Cr^3+^ ions from the MSNPs–CAAQ surface.

A suitable desorbing agent was needed for the quantitative recovery of the adsorbed Cr^3+^ ions onto MSNPs–CAAQ. For this purpose, several desorbing agents with different concentrations, including HCl, HNO_3_, and Na_2_EDTA, were examined and are shown in Figure 16a–c, respectively. As observed in Figure 16a,b, the use of HCl and HNO_3_ with concentrations of less than 0.10 mol/L did not quantitatively recover the retained Cr^3+^ ion. The higher concentration of HCl and HNO_3_ (0.20 mol/L) was effective, and HCl with a concentration of 0.20 mol/L was more effective than the same concentration, as well as HNO_3_; the desorption for Cr^3+^ ions could reach 95.6% for the first cycle. When using Na_2_EDTA as a desorbing agent in Figure 16c, the higher concentration above 0.10 mol/L was more effective than the low concentration of 0.05 mol/L, and the desorption for Cr^3+^ ions could reach 97.5%. Therefore, based on the experimental results and economic considerations, 0.2 mol/L HCl, 0.2 mol/L HNO_3_, and 0.1 mol/L Na_2_EDTA were selected as the desorbing agents to desorpt HMIs on the surface of MSNPs–CAAQ.

**Figure 17:**
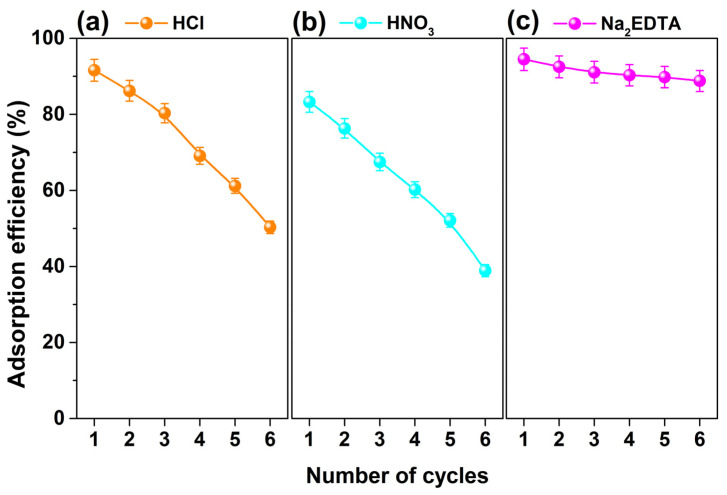
Effect of adsorbent regeneration times on the adsorption efficiencies using (**a**) 0.2 mol/L HCl, (**b**) 0.2 mol/L HNO_3_, and (**c**) 0.1 mol/L Na_2_EDTA as desorbing agents. (Initial adsorbent was 1.0 g/L; Cr^3+^ concentration was 200 mg/L; *V* = 50 mL; desorption time was 12 h; re–adsorption time was 1.5 h; 298.15 K).

The adsorption–desorption experiments were conducted using HCl, HNO_3_, and Na_2_EDTA as the desorbing agents for six successive cycles at room temperature, and the regenerated MSNPs–CAAQ was reused in the next cycle of adsorption experiments. Figure 17a–c shows the regeneration plot of MSNPs–CAAQ for the re–adsorption of Cr^3+^ ions using HCl (0.2 mol/L), HNO_3_ (0.2 mol/L), and Na_2_EDTA (0.1 mol/L) as desorbing agents, respectively. When using 0.2 mol/L HCl and 0.2 mol/L HNO_3_ as desorbing agents, as shown in Figure 17a,b, the adsorption efficiencies of Cr^3+^ ions on regenerated MSNPs–CAAQ were 91.6% and 83.3% in the first cycle and reduced to 50.3% and 38.9% after six consecutive regeneration cycles. These results indicated that neither HCl nor HNO_3_ were suitable desorbing agents for HMIs–loaded MSNPs–CAAQ, which could be due to the deformation effects on the MSNPs–CAAQ sorbent surface during desorption and the mass loss caused by demagnetization. When 0.1 mol/L Na_2_EDTA was used as an adsorbing agent, as shown in Figure 17c, the adsorption efficiency of the first regenerated MSNPs–CAAQ for Cr^3+^ ions reached 94.5%. Moreover, there was a certain degree of decline, but the removal efficiency for Cr^3+^ ions was maintained at 88.8% after six cycles, implying that the as–synthesized MSNPs–CAAQ nano–sorbent has a great economic value.

## 3. Experimental Procedure

### 3.1. Starting Materials

FeCl_2_·4H_2_O (AR), FeCl_3_·6H_2_O (AR), tetraethyl orthosilicate (TEOS, >99.0%), 3–chloropropyltrichlorosilane (CPTCS, >97.0%), triethylamine (99%), and EDTA disodium salt dihydrate (Na_2_EDTA, 99.999%) were obtained from Shanghai Macklin Biochemical Co., Ltd. (Shanghai, China). Methyltrichlorosilane (MTCS, >98.0%) and branched polyethylenimine (PEI, Mw ~25,000) were obtained from Tokyo (Shanghai, China) Chemical Industry Co., Ltd. (Shanghai, China) and Sigma–Aldrich (Shanghai, China) Trading Co., Ltd. (Shanghai, China), respectively. Ethanol, hexane, dichloromethane, acetonitrile, NH_3_·H_2_O (25~28 wt.%), and 8–aminoquinoline (98%) were purchased from Chengdu Kelong Chemical Co., Ltd. (Chengdu, China) and Shanghai Civi Chemical Technology Co., Ltd. (Shanghai, China), respectively. Chloroacetyl chloride (98%) was purchased from Shanghai Xianding Biological Science and Technology Co., Ltd. (Shanghai, China).

### 3.2. Synthesis

#### 3.2.1. Synthesis of Core–Shell MSNPs

Firstly, MNPs with hydroxyl groups (–OH) were synthesized based on a simple chemical co–precipitation method. FeCl_2_·4H_2_O (10 mmol) and FeCl_3_∙6H_2_O (20 mmol) were dissolved into 120 mL of distilled water under an N_2_ atmosphere with mechanical stirring, to which 60 mL of NH_3_·H_2_O was added; then, the mixture was heated to 70 °C and left for 30 min. After that, the precipitate was washed with distilled water and dispersed into a solution of sodium citrate dihydrate (150 mL, 20 mmol/L) with mechanical stirring under an N_2_ atmosphere for 12 h at room temperature. Subsequently, the citrate–modified MNPs were washed with distilled water and ethanol.

**Figure 18:**
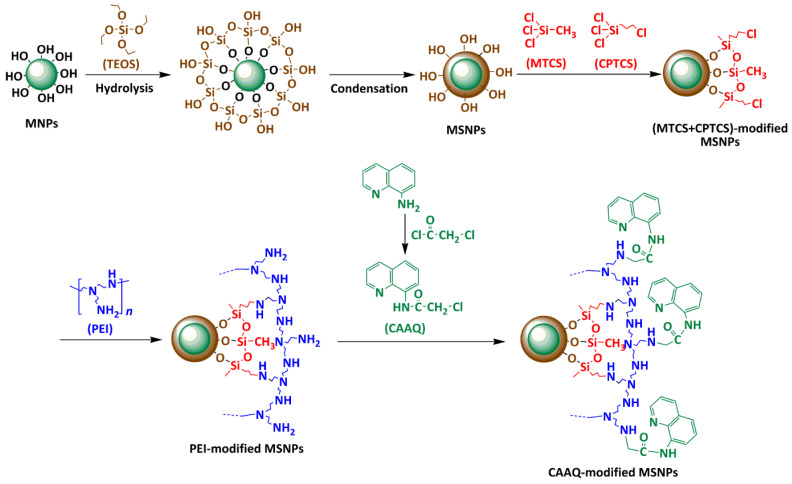
Schematic diagram illustrating the processes of the synthesis of MSNPs and surface modification with CAAQ onto MSNPs via a combined “grafting–from” and “grafting–to” approach; 3.3. synthesis of MSNPs–CAAQ using a three–step grafting strategy.

Following that, the core–shell MSNPs were synthesized via hydrolysis and the condensation of TEOS onto the MNPs’ surface, as shown in Figure 18. A total of ~0.56 g citrate–modified MNPs and 5.0 mL NH_3_·H_2_O were added to 120 mL ethanol under an N_2_ atmosphere with mechanical stirring; then, 4.0 mL TEOS was added, and the mixture was left for 8 h at room temperature. After this reaction, the as–synthesized MSNPs were washed with ethanol.

The core–shell MSNPs were modified with CAAQ to fabricate the network polymer coating through a three–step grafting strategy involving MTCS/CPTCS’s co–modification, PEI–grafting, and CAAQ–grafting. As shown in Figure 18, the as–synthesized MSNPs were first modified with MTCS and CPTCS to yield chlorine groups, which provided the preconditions for subsequent surface modifications. Following that, the branched PEI was grafted onto chlorine–modified MSNPs to equip them with amino–active functional groups. Finally, the pre–synthesized CAAQ was immobilized onto MSNPs–PEI through the coupling reactions between the amines and chlorine groups.

#### 3.2.2. Synthesis of MTCS and CPTCS co–Modified MSNPs

MSNPs (~0.5 g), MTCS (17.6 mmol), and CPTCS (1.4 mmol) were added to 150 mL of hexane, and the mixture was left for 24 h at room temperature in a hydrochloric acid atmosphere. After that, the as–synthesized MTCS and CPTCS co–modified MSNPs (labeled as MSNPs–MTCS/CPTCS) were washed with hexane, ethanol, and distilled water.

#### 3.2.3. Synthesis of PEI–Modified MSNPs

The MSNPs–MTCS/CPTCS, PEI (1.0 mL), and methanol (5.0 mL) mentioned above were added to 120 mL of distilled water with mechanical stirring under an N_2_ atmosphere, and the mixture was left for 48 h at 65 °C. After that, the as–synthesized PEI–modified MSNPs (labeled as MSNPs–PEI) were washed with distilled water.

#### 3.2.4. Pre–Synthesis of CAAQ and Synthesis of CAAQ–Modified MSNPs by “Grafting–to” Approach

CAAQ can be pre–synthesized by a simple organic reaction. Briefly, 16.4 mmol 8–aminoquinoline and 2.5 mL triethylamine were mixed in 100 mL dichloromethane in an ice bath with magnetic stirring for 30 min in darkness. Then, 14.9 mmol of chloroacetyl chloride was added, and the mixture was left for 48 h at room temperature. After reaction, the crude products (8–chloroacetyl–aminoquinoline (CAAQ)) were collected by evaporating the solvent and purified by silica column chromatography (*V*_Petroleum ether_/*V*_Ethylacetate_ = 3/1).

Finally, the pre–synthesized CAAQ was securely immobilized onto MSNPs–PEI through a series of coupling reactions between the amines (–NH_2_) and chlorine (–Cl) groups. The PEI–modified MSNPs discussed above and 5.4 mmol CAAQ were added to 110 mL acetonitrile with mechanical stirring, and the mixture was refluxed at 60 °C for 24 h under N_2_ atmosphere. After further aging for 24 h, the CAAQ–modified MSNPs (labeled as MSNPs–CAAQ) were collected and washed with acetonitrile and ethanol.

### 3.3. Characterization

The crystallographic phases of samples were characterized by X–ray diffraction (XRD, DX–2700, Dandong, China). The morphology and size of samples were examined by transmission electron microscopy (TEM, JEM–2100, Tokyo, Japan). Fourier transform infrared spectra (FTIR) of samples were recorded on a Nicolet iS10 spectrometer (Thermo Scientific, Waltham, MA, USA). X–ray photoelectron spectroscopy (XPS) analyses of samples were performed using an ESCALAB 250Xi spectrometer (Thermo Scientific, Waltham, MA, USA). Thermogravimetric (TG) curves of samples were collected by an SDT–2960 thermogravimetric instrument (TA Instruments, New Castle, DE, USA) at a heating rate of 10 °C/min under a flow of air. The quantitative analyses of HMIs in the adsorption and desorption experiments were performed using atomic absorption spectrometry (AAS, AA–6800, Shimadzu, Japan) and inductively coupled plasma mass spectrometry (ICP–MS, VG PQ ExCell, Thermo Electron Co., Vallejo, CA, USA). The magnetic properties of samples were obtained using a vibrating sample magnetometer (VSM, Lakeshore 7307, Novi, MI, USA) at room temperature.

### 3.4. Adsorption Experiments

The adsorption characteristics of MSNPs–CAAQ nanocomposites were evaluated by removing HMIs from simulated wastewaters; the selected HMIs included Ag^+^, Zn^2+^, Cu^2+^, Co^2+^, Cd^2+^, Mn^2+^, Pb^2+^, Hg^2+^, Cr^3+^ and Fe^3+^ ions. A total of 0.05 g MSNPs–CAAQ was dispersed into the set HMIs aqueous solution (50 mL, 50~500 mg/L). The mixture was stirred at a constant speed of 200 rpm at a set temperature of 298.15~338.15 K. After the adsorption reaction occurred within a given time (*t*, *t* = 0~2.5 h), the particles were collected by magnetic separation, and the concentrations of these HMIs in the supernatant were measured using ICP–MS. The adsorption efficiency at time *t* (*A*_t_, %) and equilibrium adsorption amount (*q*_m_, mg/g) of HMIs were calculated using Equation (1) and Equation (2), respectively.

(1) $$A_{t}=\frac{C_{0}-C_{t}}{C_{0}}\times 100\%$$

(2) $$q_{e}=\frac{(C_{0}-C_{e})V}{m}$$

where *C*_0_ (mg/L) is the initial concentration of HMIs’ aqueous solution, *C*_t_ (mg/L) is the concentration of HMIs at the given time *t*, *m* (g) is the mass of MSNPs–CAAQ, and *V* (L) is the volume of the HMIs aqueous solution.

To investigate the adsorption mechanism between MSNPs–CAAQ and HMIs, the experimental data were analyzed by the Langmuir isotherm model and the Freundlich isotherm model using Equation (3) [58] and Equation (4) [59], respectively:

(3) $$\frac{C_{e}}{q_{e}}=\frac{1}{q_{m}}C_{e}+\frac{1}{K_{L}q_{m}}$$

(4) $$\log q_{e}=\frac{1}{n}\log C_{e}+\log K_{F}$$

where *C*_e_ (mg/L), *q*_e_ (mg/g), and *q*_m_ (mg/g) are the equilibrium concentration, equilibrium quantity, and saturated adsorption amounts, respectively. *K*_L_ and *K*_F_ are the Langmuir adsorption constants related to the affinity of the binding site and the Freundlich adsorption constants related to the adsorption capacity, respectively, and *n* is the adsorption intensity. Moreover, *q*_m_ and *K*_L_ can be evaluated by the plot of *C*_e_/*q*_e_ against *C*_e_, while *n* and *K*_F_ can be evaluated by the plot of log (*q*_e_) against log (*C*_e_).

To explore the thermodynamic characteristics of the adsorption process, the enthalpy change (Δ*H*^0^, KJ/mol) and entropy change (Δ*S*^0^, J/mol·K) were calculated using the Van’t Hoff plot using Equation (5) [60]. To investigate the kinetic characteristics of the adsorption reaction, the experimental data of adsorption were evaluated by the pseudo–first–order and pseudo–second–order models using Equations (6) and (7) [61], respectively.

(5) $$\log\frac{q_{e}}{C_{e}}=-\frac{\Delta H^{0}}{2.303R}\times \frac{1}{T}+\frac{\Delta S^{0}}{2.303R}$$

where *T* (K) is the temperature of the adsorption reaction; *R* (J/mol·K) is the gas constant per molecule.

(6) $$\log(q_{e1,\mathrm{cal}}-q_{t})=-\frac{k_{1}}{2.303}t+\log q_{e1,\mathrm{cal}}$$

where *q*_e1, cal_ (mg/L), and *k*_1_ (1/h) are the calculated equilibrium adsorption amount and the rate constant of the pseudo–first–order equation, respectively, which can be evaluated by the plot of log(*q*_e1,cal_–*q*_t_) against *t*:

(7) $$\frac{t}{q_{t}}=\frac{1}{q_{e2,\mathrm{cal}}}t+\frac{1}{k_{2}q_{e2,\mathrm{cal}}^{2}}$$

where *q*_e2,cal_ (mg/L), and *k*_2_ (g/mg·h) are the calculated equilibrium adsorption amount and the rate constant of the pseudo–second–order equation, respectively, which can be evaluated by the plot of *t*/*q*_t_ against *t*.

### 3.5. Desorption and Reusability Experiments

In order to regenerate MSNPs–CAAQ nanocomposites, an elution step using a suitable desorbing agent (HCl, HNO_3_, or Na_2_EDTA) was carried out after each adsorption cycle. After the adsorption of the saturated HMIs, the HMIs–loaded MSNPs–CAAQ was exposed to HCl, HNO_3_, or Na_2_EDTA solution with a desired concentration to allow for regeneration for 12 h under continuous mechanical stirring and was then magnetically separated from the water sample and washed several times with distilled water. The regenerated MSNPs–CAAQ were reused in the next cycle of adsorption experiments, and the adsorption–desorption experiments were conducted for six cycles at room temperature.

## 4. Conclusions

In summary, we presented a general strategy to synthesize network–polymer–modified magnetic nanomaterials. First, we designed and synthesized core–shell MSNPs with a ~10 nm core and ~5.5 nm shell through the hydrolysis and condensation of TEOS onto a single–crystal Fe_3_O_4_ seed. Following that, the core–shell MSNPs were modified with CAAQ to fabricate the network polymer coating through a three–step grafting strategy, in which polymer grafting was designed using a macromolecular polymer (PEI) to fabricate an organic network onto the MSNPs surface and to serve as a bridge between the surface hydroxyl group and CAAQ. The as–synthesized MSNPs–CAAQ nanocomposites showed a strong magnetic response under an external magnet, which made it easier to recycle quickly in practical applications. The adsorption experimental results indicated that MSNPs–CAAQ had an excellent absorption capacity for HMIs due to their abundant end–reactive functional groups: the saturated adsorption amounts of Fe^3+^, Cu^2+^ and Cr^3+^ ions were 324.7, 306.8, and 293.3 mg/g, respectively, according to the Langmuir linear fitting. Moreover, MSNPs–CAAQ also exhibited universal adsorption capacities for Zn^2+^, Mn^2+^, and Pb^2+^ ions, with adsorption efficiencies over 70%. The adsorption isotherm for the Cr^3+^ ion fitted the experimental data well when using the Langmuir isotherm model, and its adsorption kinetics could be described by the pseudo–second–order equation. More importantly, Na_2_EDTA (0.10 mol/L) was more suitable as a desorbing agent for MSNPs–CAAQ regeneration than HCl (0.20 mol/L) and HNO_3_ (0.20 mol/L). Additionally, the adsorption efficiency of the Cr^3+^ ion on regenerated MSNPs–CAAQ could still be maintained at 88.8% after six adsorption–desorption cycles, showing the high economic value of the as–synthesized MSNPs–CAAQ nano–sorbent. Moreover, our design provides a general strategy for the fabrication of high–performance HMIs absorbents and can easily be extended to other absorption materials for actual applications.

## Figures and Tables

**Table 1:** Relevant parameters of Langmuir isotherms for the adsorption of Fe^3+^, Cu^2+^, and Cr^3+^ ions.

Langmuir Isotherm Model	$\frac{C_{e}}{q_{e}}=\frac{1}{q_{m}}C_{e}+\frac{1}{K_{L}q_{m}}$		
Parameters	*q*_m_ (mg/g)	*K*_L_	*R* ^2^
Fe^3+^	324.7	0.7016	0.9997
Cu^2+^	306.8	0.6981	0.9988
Cr^3+^	293.3	0.7894	0.9990

**Table 2:** Comparison of maximum adsorption capacities of various adsorbents for Fe^3+^, Cu^2+^, and Cr^3+^ ions.

Adsorbent	HMIs	Adsorption Capacity (*q*_m_, mg/g)	Reference
Manganese dioxide–modified biochar	Fe^3+^	52.39	[47]
Activated biochars prepared by leucaena leucocephala	Fe^3+^	32.89	[48]
Phosphorylated nanocellulose (Phos–CNCSL)	Fe^3+^	115	[49]
	Cu^2+^	117	
Fe_3_O_4_/FeMoS_4_/MgAl–LDH nanocomposite	Cu^2+^	108.28	[50]
Core–shell magnetic Fe_3_O_4_@zeolite NaA	Cu^2+^	86.54	[51]
EDTA–inspired polydentate hydrogels	Cu^2+^	436.5	[52]
	Cr^3+^	340.6	
Organosulphur–modified biochar	Cr^3+^	35.2	[53]
Porous carbon materials derived from rice wastes	Cr^3+^	9.23	[54]
MSNPs–CAAQ nanocomposite	Fe^3+^	324.7	This work
	Cu^2+^	306.8	
	Cr^3+^	293.3	

**Table 3:** Thermodynamic parameters for the adsorption of Cr^3+^ ions onto MSNPs–CAAQ.

Van’t Hoff Thermodynamic Equation	$\log\frac{q_{e}}{c_{e}}=-\frac{\Delta H^{0}}{2.303R}\times \frac{1}{T}+\frac{\Delta S^{0}}{2.303R}$		
**Parameters**	**Δ*H*^0^ (KJ/mol)**	**Δ*S*^0^ (J/mol·K)**	***R* ^2^**
	2.31	45.00	0.9993

**Table 4:** Kinetic parameters for the adsorption of Cr^3+^ ions onto MSNPs–CAAQ surfaces.

Kinetic Model	Pseudo–First–Order $\log(q_{e1,\mathrm{cal}}-q_{t})=-\frac{k_{1}}{2.303}t+\log q_{e1,\mathrm{cal}}$			Pseudo–Second–Order $\frac{t}{q_{t}}=\frac{1}{q_{e2,\mathrm{cal}}}t+\frac{1}{k_{2}q_{e2,\mathrm{cal}}^{2}}$		
**Parameters**	***q*_e1,cal_ (mg/g)**	***K*_1_ (1/h)**	***R* ^2^**	***q*_e2,cal_ (mg/g)**	***K*_1_ (g/mg·h)**	***R* ^2^**
	93.61	1.8801	0.9841	206.61	0.03839	0.9989

## Data Availability

Not applicable.

## References

[1] Su X., Kushima A., Halliday C., Zhou J., Li J., Hatton T.A. (2018). Electrochemically–mediated selective capture of heavy metal chromium and arsenic oxyanions from water. Nat. Commun..

[2] Li B., Zhang Y., Ma D., Shi Z., Ma S. (2014). Mercury nano–trap for effective, efficient removal of mercury(II) from aqueous solution. Nat. Commun..

[3] Liu C., Wu T., Hsu P.C., Xie J., Zhao J., Liu K., Sun J., Xu J., Tang J., Ye Z. (2019). Direct/alternating current electrochemical method for removing and recovering heavy metal from water using graphene oxide electrode. ACS Nano.

[4] Wu C.S., Khaing Oo M.K., Fan X. (2010). Highly sensitive multiplexed heavy metal detection using quantum–dot–labeled dnazymes. ACS Nano.

[5] Ojeajiménez I., López X., Arbiol J., Puntes V. (2012). Citrate–coated gold nanoparticles as smart scavengers for mercury(II) removal from polluted waters. ACS Nano.

[6] Salt D.E., Blaylock M., Kumar N.P.B.A., Dushenkov V., Ensley B.D., Chet I., Raskin I. (1995). Phytoremediation: A novel strategy for the removal of toxic metals from the environment using plants. Nat. Biotechnol..

[7] Kumar R., Patil S.A. (2020). Removal of heavy metals using bioelectrochemical systems. Integrated Microbial Fuel Cells for Wastewater Treatment.

[8] Kumar R., Yadav S., Patil S.A. (2020). Bioanode–Assisted Removal of Hg^2+^ at the Cathode of Microbial Fuel Cells. J. Hazard. Toxic Radio..

[9] Nazir M.A., Bashir M.A., Najam T., Javad M.S., Suleman S., Hussain S., Kumar O.P., Shah S.S.A., Rehman A.U. (2021). Combining structurally ordered intermetallic nodes: Kinetic and isothermal studies for removal of malachite green and methyl orange with mechanistic aspects. Microchem. J..

[10] Rauwel P., Rauwel E. (2019). Towards the Extraction of Radioactive Cesium-137 from Water via Graphene/CNT and Nanostructured Prussian Blue Hybrid Nanocomposites: A Review. Nanomaterials.

[11] Zhang Y., Niu Q., Gu X., Yang N., Zhao G. (2019). Recent progress of carbon nanomaterials for electrochemical detection and removal of environmental pollutants. Nanoscale.

[12] Kumar R., Rauwel P., Rauwel E. (2021). Nanoadsorbants for the Removal of Heavy Metals from Contaminated Water: Current Scenario and Future Directions. Processes.

[13] Jha V.K., Nagae M., Matsuda M., Miyake M. (2009). Zeolite formation from coal fly ash and heavy metal ion removal characteristics of thus–obtained Zeolite X in multi–metal systems. J. Environ. Manag..

[14] Efome J.E., Rana D., Matsuura T., Lan C.Q. (2018). Metal–organic frameworks supported on nanofibers to remove heavy metals. J. Mater. Chem. A.

[15] Yan H., Li H., Tao X., Li K., Yang H., Li A., Xiao S., Cheng R. (2014). Rapid Removal and Separation of Iron(II) and Manganese(II) from Micropolluted Water Using Magnetic Graphene Oxide. ACS Appl. Mater. Interfaces.

[16] Bolisetty S., Peydayesh M., Mezzenga R. (2019). Sustainable technologies for water purification from heavy metals: Review and analysis. Chem. Soc. Rev..

[17] Zhao W., Chen I.W., Huang F. (2019). Toward large–scale water treatment using nanomaterials. Nano Today.

[18] Kumar R., Rauwel P., Kriipsalu M., Wragg D., Rauwel E. (2023). Nanocobalt based (Co@Co(OH)_2_) sand nanocomposite applied to manganese extraction from contaminated water. J. Environ. Chem. Eng..

[19] Kulpa–Koterwa A., Ossowski T., Niedziałkowski P. (2021). Functionalized Fe_3_O_4_ Nanoparticles as Glassy Carbon Electrode Modifiers for Heavy Metal Ions Detection—A Mini Review. Materials.

[20] Lisjak D., Mertelj A. (2018). Anisotropic magnetic nanoparticles: A review of their properties, syntheses and potential applications. Prog. Mater. Sci..

[21] Han X., Deng Z., Yang Z., Wang Y., Zhu H., Chen B., Cui Z., Ewing R., Shi D. (2017). Biomarkerless targeting and photothermal cancer cell killing by surface–electrically–charged superparamagnetic Fe_3_O_4_ composite nanoparticles. Nanoscale.

[22] Young K.L., Xu C., Xie J., Sun S. (2009). Conjugating methotrexate to magnetite (Fe_3_O_4_) nanoparticles via trichloro–s–triazine. J. Mater. Chem..

[23] Liu S., Guo S., Sun S., You X.Z. (2015). Dumbbell–like Au–Fe_3_O_4_ nanoparticles: A new nanostructure for supercapacitors. Nanoscale.

[24] Shi X., You W.B., Zhao Y., Li X., Shao Z., Che R. (2019). Multi–scale magnetic coupling of Fe@SiO_2_@C–Ni yolk@triple–shell microsphere for broadband microwave absorption. Nanoscale.

[25] Ni Q., Chen B., Dong S., Tian L., Bai Q. (2015). Preparation of core–shell structure Fe_3_O_4_@SiO_2_ superparamagnetic microspheres immoblized with iminodiacetic acid as immobilized metal ion affinity adsorbents for His–tag protein purification. Biomed. Chromatogr..

[26] Jin S., Park B.C., Ham W.S., Pan L., Kim Y.K. (2017). Effect of the magnetic core size of amino–functionalized Fe_3_O_4_–mesoporous SiO_2_ core–shell nanoparticles on the removal of heavy metal ions. Colloids Surf. A.

[27] Liu Y., Fu R., Sun Y., Zhou X., Baig S.A., Xu X. (2016). Multifunctional nanocomposites Fe_3_O_4_@SiO_2_–EDTA for Pb(II) and Cu(II) removal from aqueous solutions. Appl. Surf. Sci..

[28] Pogorilyi R., Pylypchuk I., Melnyk I., Zub Y., Seisenbaeva G., Kessler V. (2017). Sol–gel derived adsorbents with enzymatic and complexonate functions for complex water remediation. Nanomaterials.

[29] Zhang S., Zhang Y., Liu J., Xu Q., Xiao H., Wang X., Xu H., Zhou J. (2013). Thiol modified Fe_3_O_4_@SiO_2_ as a robust, high effective, and recycling magnetic sorbent for mercury removal. Chem. Eng. J..

[30] Chen Y., Xiong Z., Zhang L., Zhao J., Zhang Q., Peng L., Zhang W., Ye M., Zou H. (2015). Facile synthesis of zwitterionic polymer–coated core–shell magnetic nanoparticles for highly specific capture of N–linked glycopeptides. Nanoscale.

[31] Li S., Li N., Yang S., Liu F., Zhou J. (2014). The synthesis of a novel magnetic demulsifier and its application for the demulsification of oil–charged industrial wastewaters. J. Mater. Chem. A.

[32] Liu G., Cai M., Wang X., Zhou F., Liu W. (2014). Core–shell–corona–structured polyelectrolyte brushes–grafting magnetic nanoparticles for water harvesting. ACS Appl. Mater. Inter..

[33] Khan A.A., Khan I.A., Siyal M.I., Lee C.K., Kim J.O. (2019). Optimization of membrane modification using SiO_2_ for robust anti–fouling performance with calcium–humic acid feed in membrane distillation. Environ. Res..

[34] Ruhi G., Bhandari H., Dhawan S.K. (2014). Designing of corrosion resistant epoxy coatings embedded with polypyrrole/SiO_2_ composite. Prog. Org. Coat..

[35] Chen Z., Wang J., Pu Z., Zhao Y., Jia D., Chen H., Wen T., Hu B., Alsaedi A., Hayat T. (2017). Synthesis of magnetic Fe_3_O_4_/CFA composites for the efficient removal of U(VI) from wastewater. Chem. Eng. J..

[36] Duffy E., Mitev D.P., Thickett S.C., Townsend A.T., Paull B., Nesterenko P.N. (2015). Assessing the extent, stability, purity and properties of silanised detonation nanodiamond. Appl. Surf. Sci..

[37] Zhang S., Kai C., Liu B., Zhang S., Wei W., Xu X., Zhou Z. (2020). Facile fabrication of cellulose membrane containing polyiodides and its antibacterial properties. Appl. Surf. Sci..

[38] Karimi Shervedani R., Rezvaninia Z., Sabzyan H. (2015). Oxinate–aluminum(III) nanostructure assemblies formed via in–situ and ex–situ oxination of gold–self–assembled monolayers characterized by electrochemical, attenuated total reflectance fourier transform infrared spectroscopy, and X–ray photoelectron spectroscopy methods. Electrochim. Acta.

[39] Beard B.C. (1990). Cellulose nitrate as a binding energy reference in N(1s) XPS studies of nitrogen–containing organic molecules. Appl. Surf. Sci..

[40] Liu H., Zhou Y., Yang Y., Zou K., Wu R., Xia K., Xie S. (2019). Synthesis of polyethylenimine/graphene oxide for the adsorption of U(VI) from aqueous solution. Appl. Surf. Sci..

[41] El–Rahman H.A.A., Schultze J.W. (1996). New quaternized aminoquinoline polymer films: Electropolymerization and characterization. J. Electroanal. Chem..

[42] Jackson S. (1995). Determining hybridization differences for amorphous carbon from the XPS C 1s envelope. Appl. Surf. Sci..

[43] Choi S.J., Kim S.J., Kim I.D. (2016). Ultrafast optical reduction of graphene oxide sheets on colorless polyimide film for wearable chemical sensors. NPG Asia Mater..

[44] Pan N., Li L., Ding J., Wang R., Jin Y., Xia C. (2017). A schiff base/quaternary ammonium salt bifunctional graphene oxide as an efficient adsorbent for removal of Th(IV)/U(VI). J. Colloid Interface Sci..

[45] Kosa S.A., Al–Zhrani G., Abdel Salam M. (2012). Removal of heavy metals from aqueous solutions by multi–walled carbon nanotubes modified with 8–hydroxyquinoline. Chem. Eng. J..

[46] Lv Z.P., Luan Z.Z., Cai P.Y., Wang T., Li C.H., Wu D., Zuo J.L., Sun S. (2016). Enhancing magnetoresistance in tetrathiafulvalene carboxylate modified iron oxide nanoparticle assemblies. Nanoscale.

[47] Maneechakr P., Karnjanakom S. (2019). Environmental surface chemistries and adsorption behaviors of metal cations (Fe^3+^, Fe^2+^, Ca^2+^ and Zn^2+^) on manganese dioxide–modified green biochar. RSC Adv..

[48] Maneechakr P., Karnjanakom S. (2019). The essential role of Fe(III) ion removal over efficient/low–cost activated carbon: Surface chemistry and adsorption behavior. Res. Chem. Intermed..

[49] Liu P., Borrell P.F., Božič M., Kokol V., Oksman K., Mathew A.P. (2015). Nanocelluloses and their phosphorylated derivatives for selective adsorption of Ag^+^, Cu^2+^ and Fe^3+^ from industrial effluents. J. Hazard. Mater..

[50] Behbahani E.S., Dashtian K., Ghaedi M. (2021). Fe_3_O_4_–FeMoS_4_: Promise magnetite LDH–based adsorbent for simultaneous removal of Pb (II), Cd (II), and Cu (II) heavy metal ions. J. Hazard. Mater..

[51] Cao J., Wang P., Shen J., Sun Q. (2020). Core–shell Fe_3_O_4_@zeolite NaA as an adsorbent for Cu^2+^. Materials.

[52] Panja S., Hanson S., Wang C. (2020). EDTA–inspired polydentate hydrogels with exceptionally high heavy metal adsorption capacity as reusable adsorbents for wastewater purification. ACS Appl. Mater. Interfaces.

[53] Macedo J.C.A., Gontijo E.S.J., Herrera S.G., Rangel E.C., Komatsu D., Landers R., Rosa A.H. (2021). Organosulphur–modified biochar: An effective green adsorbent for removing metal species in aquatic systems. Surf. Interfaces.

[54] Dias D., Bernardo M., Matos I., Fonseca I., Pinto F., Lapa N. (2020). Activation of co–pyrolysis chars from rice wastes to improve the removal of Cr^3+^ from simulated and real industrial wastewaters. J. Clean. Prod..

[55] Deraeve C., Maraval A., Vendier L., Faugeroux V., Pitié M., Meunier B. (2008). Preparation of new bis(8–aminoquinoline) ligands and comparison with bis(8–hydroxyquinoline) ligands on their ability to chelate Cu^II^ and Zn^II^, Eur. J. Inorg. Chem..

[56] Huang J., Xu Y., Qian X. (2014). Rhodamine–based fluorescent off–on sensor for Fe^3+^–in aqueous solution and in living cells: 8–aminoquinoline receptor and 2:1 binding. Dalton Trans..

[57] Mirzaei M., Eshtiagh–Hosseini H., Bolouri Z., Rahmati Z., Esmaeilzadeh A., Hassanpoor A., Bauza A., Ballester P., Mague J.T., Notash B. (2015). Rationalization of noncovalent interactions within six new MII/8–aminoquinoline supramolecular complexes (MII = Mn, Cu, and Cd): A combined experimental and theoretical DFT study. Cryst. Growth Des..

[58] Langmuir I. (1918). The adsorption of gases on plane surfaces of glass, mica and platinum. J. Am. Chem. Soc..

[59] Freundlich H. (1907). Uber die adsorption in lo sungen. J. Phys. Chem..

[60] Das S., Mishra S. (2020). Insight into the isotherm modelling, kinetic and thermodynamic exploration of iron adsorption from aqueous media by activated carbon developed from *Limonia acidissima* shell. Mater. Chem. Phys..

[61] Schiewer S., Patil S.B. (2008). Pectin–rich fruit wastes as biosorbents for heavy metal removal: Equilibrium and kinetics. Bioresour. Technol..

